# Excitation of filamentous growth in *Dekkera* spp. by quorum sensing aromatic alcohols 2-phenylethanol and tryptophol

**DOI:** 10.1093/femsle/fnae105

**Published:** 2024-12-05

**Authors:** Scott J Britton, Thijs Dingemans, Lisa J Rogers, Jane S White, Dawn L Maskell

**Affiliations:** International Centre for Brewing and Distilling, Institute of Biological Chemistry, Biophysics, and Bioengineering, School of Engineering and Physical Sciences, Heriot-Watt University, Edinburgh EH14 4AS, United Kingdom; Research and Development, Brouwerij Duvel Moortgat, 2870 Puurs-Sint-Amands, Belgium; Research and Development, Brouwerij Duvel Moortgat, 2870 Puurs-Sint-Amands, Belgium; TranscendED, Brooklyn, NY 11232 United States; International Centre for Brewing and Distilling, Institute of Biological Chemistry, Biophysics, and Bioengineering, School of Engineering and Physical Sciences, Heriot-Watt University, Edinburgh EH14 4AS, United Kingdom; International Centre for Brewing and Distilling, Institute of Biological Chemistry, Biophysics, and Bioengineering, School of Engineering and Physical Sciences, Heriot-Watt University, Edinburgh EH14 4AS, United Kingdom

**Keywords:** quorum sensing, sociomicrobiology, aromatic alcohols, pseudohyphae, *Brettanomyces*, fungi

## Abstract

Fungi from the genus *Dekkera*, also known as *Brettanomyces*, are significant contaminants in commercial beer and wine production, and when present unintentionally, these non-domesticated yeasts result in the development of undesirable sensorial characteristics, in part due to the production of volatile phenols and acetate esters. The persistence of *Dekkera* spp. in industrial manufacturing environments can be attributed to its strong bioadhesive properties, allowing it to attach to various surfaces and form biofilms, which often contribute to recurrent contaminations. In other fungi, the yeast-to-filamentous transition is pivotal in enhancing bioadhesive properties, a process tightly regulated by density-dependent quorum-sensing mechanisms. However, there is no documented evidence regarding the influence of fungal quorum-sensing compounds on the yeast-to-filamentous transition in *Dekkera*, nor is there any evidence of existing quorum-sensing circuits in this genus. In this investigation, two *Dekkera* spp. were cultivated on a modified nitrogen-limiting synthetic low-ammonium dextrose medium supplemented with exogenous concentrations of quorum-sensing molecules 2-phenylethanol and tryptophol. Following cultivation, whole colonies were imaged and analyzed with a whole colony filamentation algorithm to quantify their filamentation. Our results demonstrate that the quorum-sensing compounds 2-phenylethanol and tryptophol significantly promote the yeast-to-filamentous transition in *Dekkera* spp., underscoring the broader presence of quorum-regulated social behaviors within this genus.

## Teaser

This research demonstrates that quorum-sensing compounds 2-phenylethanol and tryptophol significantly promote the yeast-to-filamentous transition in *Dekkera* spp., revealing the presence of quorum-regulated social behaviors in this fungal genus.

## Introduction

When inadvertently introduced into beer, wine, cider, dairy products, or other beverages, yeasts from the genus *Dekkera* (teleomorphic form) belonging to the family *Ascomycota*, commonly recognized as *Brettanomyces* (anamorphic form), cause turbidity and produce high levels of undesirable off-flavors, such as volatile phenols and acetate esters, leading to unwanted sensorial features often described as horse sweat, barnyard, medicinal, leathery, or even mousey (Chatonnet et al. 1992, Steensels et al. 2015, Thompson-Witrick and Pitts 2022, Dusart et al. 2023, Le Montagner et al. 2024b, Lentz 2024, Ogata and Saito 2024). However, it is essential to acknowledge that the sensory characteristics imparted by *Dekkera* spp., while often regarded as undesirable in certain brewing contexts, are also highly sought after in specific beer styles. The distinct and complex profile of flavors and aromas are integral to the identity of traditional and artisanal beers such as Lambics, Gueuze, and some farmhouse ales (Verachtert and Derdelinckx 2014, Bossaert et al. 2019, De Roos et al. 2019, Shayevitz et al. 2020, Thompson-Witrick and Pitts 2022, Dusart et al. 2023).

These yeast exhibit a heightened capacity to colonize cellars; contaminate pumps, pipes, and manufacturing equipment; and, most critically, form robust biofilms, earning its reputation as one of the most severe beverage spoilage organisms, often enduring for prolonged periods on biotic or abiotic reservoirs, thereby facilitating the transfer of these wild yeast from the reservoir into fermentation processes (Bojsen et al. 2012, Cibrario et al. 2019, Lebleux et al. 2020, Dimopoulou et al. 2021, Le Montagner et al. 2024b). Prior research on *Dekkera bruxellensis*, one of the five recognized species of *Dekkera*, has revealed its remarkable ability to adhere to a diverse set of materials, including glass, stainless steel, polystyrene, and wood; materials all commonly found in breweries, wineries, or other beverage production facilities (Joseph et al. 2007, Kregiel et al. 2018, De Roos et al. 2019, Lebleux et al. 2020, Bose et al. 2021, Dimopoulou et al. 2021, Le Montagner et al. 2024a).

Broadly stated, the adherence of ascomycete yeast to a surface initiates the start of biofilm formation, where specialized adhesion molecules, often 600–2500 residue mannoproteins covalently bound to cell wall glucan via modified glycosylphosphatidylinositol (GPI) anchors, facilitate interactions occurring through amyloid-like protein–protein or hydrophobic mechanisms (Lipke 2018). Upon attaching to a surface, such as the inner wall of a wooden aging barrel or stainless steel vessel used for wine or beer maturation, these organisms proliferate, forming filamentous structures, like the pseudohyphae observed in *Dekkera* species, and begin to accumulate an extracellular matrix (Vopálenská et al. 2010, De Roos et al. 2019, Shayevitz et al. 2020, Dusart et al. 2023). In other ascomycete yeasts, the transition to filamentous growth, manifested as hyphae or pseudohyphae, is associated with a marked increase in adhesion to inert surfaces, with development being directly correlated with biofilm robustness and resistance (Paramonova et al. 2009).

Quorum sensing is a genetic regulatory mechanism reliant on population density, where microorganisms utilize diffusible hormone-like signaling molecules to synchronize community-wide gene expression and cooperative social behaviors, such as the yeast-to-filamentous transition, once a critical threshold concentration of the signaling molecule in the proximate environment is reached (Hogan 2006a, Albuquerque and Casadevall 2012, Darch et al. 2012, Albuquerque et al. 2014, Britton et al. 2021, Waters and Bassler 2005, 2006b, West et al. 2012, Özkaya et al. 2017, Schuster et al. 2017, Tian et al. 2021). It is widely acknowledged that the quorum sensing mechanism strengthens the advantages offered by cooperative social behaviors, guarantees the social response yields optimal benefits when the density of cooperators is high, and curtails social behavior in lower-density environments where returns are diminished (Darch et al. 2012, Schuster et al. 2017). In other ascomycetes, like the budding yeast *Saccharomyces cerevisiae*, the volatile aromatic alcohols 2-phenylethanol and tryptophol have been demonstrated to quorum-regulate pseudohyphal growth, or similarly haploid invasive growth, under nutrient-restrictive conditions (Britton et al. 2022, Cullen and Sprague 2000, Chen and Fink 2000, 2006, 2023, Lorenz et al. 2000, Gancedo 2001, Zaman et al. 2008, 2012, Pujari and Cullen 2024). Similarly, the ascomycete *Candida albicans*, an opportunistic pathogen in humans, undergoes a morphological transition from yeast to filaments in response to the quorum-sensing compound tyrosol—a volatile aromatic alcohol, where it promotes filamentous growth during the early and intermediate stages of biofilm formation (Chen et al. 2004, Alem et al. 2006, Tian et al. 2021).

Although *Dekkera* spp. are known for their ability to form resilient biofilms, cause significant adulteration, and result in substantial economic losses across the beverage and bioethanol industries, the impact of quorum-sensing compounds on these microorganisms has yet to be explored (Steensels et al. 2015). This study explores the yeast-to-filamentous morphogenic transition, a known phenotypic characterization associated with biofilm formation, in two species of *Dekkera*—*Dekkera anomalous* and *D. bruxellensis*. The research investigates whether this morphogenic transition is quorum sensing mediated by the volatile aromatic alcohols, 2-phenylethanol and tryptophol, at concentrations up to 200 µM, which is consistent with those observed during beer, wine, and other beverage fermentations (Ayrapaa 1961, Szlavko 1973, 2008, Li et al. 2008, Zupan et al. 2013, Avbelj et al. 2015, Pereira et al. 2019).

## Materials and methods

### Strains

Two commercial *Dekkera* strains, *D. bruxellensis* (White Labs, California, USA, Cat. No. WLP650) and *D. anomalous* (White Labs, California, USA, Cat. No. WLP645). Yeast strains were delivered in a proprietary liquid medium by the commercial supplier and subsequently cultured aerobically immediately upon arrival on yeast peptone dextrose agar [2% (w/v) glucose, 2% (w/v) bacteriological peptone, 1% (w/v) yeast extract, 1.5% agar] (Sigma Aldrich, Saint Louis, Missouri, USA, Cat. No. Y1500) at 26 ± 1°C for 96 h. The four-quadrant streak technique was employed to isolate a single, discrete colony from each initial population. These two discrete colony isolates were then utilized for subsequent experiments.

### Yeast pre-culture conditions

*Dekkera* strains were pre-cultured in 150 ml of yeast peptone dextrose broth [2% (w/v) glucose, 2% (w/v) bacteriological peptone, 1% (w/v) yeast extract] (Sigma Aldrich, Saint Louis, Missouri, USA, Cat. No. Y1375), maintained aerobically and statically at 24 ± 1°C for 96 h before conducting the filamentous growth assay.

### Filamentous growth assay

Prior to inoculation on modified synthetic low-ammonium dextrose medium (mSLAD) 120 mm × 120 mm × 17 mm square agar plates [2% (w/v) dextrose (Biowest, Nuaillé, France, Cat. No. P5030), 0.17% (w/v) yeast nitrogen base without amino acids, without ammonium sulfate (Sigma Aldrich, Saint Louis, Missouri, USA, Cat. No. Y1251), 50 or 100 µM (NH_4_)_2_SO_4_ for *D. anomalous* and *D. bruxellensis*, respectively (Sigma Aldrich, Saint Louis, Missouri, USA, Cat. No.59845), 2% (w/v) agarose (VWR International, Radnor, PA, USA, Cat No. 0710), and where applicable, supplemented with 2-phenylethanol (ThermoFisher GmbH, Kandel, Germany, Cat. No. A15241) or tryptophol (ThermoFisher GmbH, Kandel, Germany, Cat. No. L02555)], cultures were serially diluted in 0.85% sterile saline solution (VWR International, Radnor, PA, USA, Cat No. X190) to 10^−3^ the original pre-culture concentration. To assess filamentation, 0.1 ml of the 10^−3^ diluted pre-culture, resulting in a 10^−4^ plated concentration, was inoculated onto four mSLAD agar plates per condition. These conditions included the two *Dekkera* strains plated on mSLAD agar plates containing varying concentrations (0, 10, 50, 100, and 200 µM) of 2-phenylethanol or tryptophol (*n* = 160). Following inoculation, the inoculum was uniformly dispersed across the surface of the mSLAD agar using a glass cell spreader to ensure consistent coverage. Plates were subsequently left upright at room temperature for 30 min, then inverted, sealed with petri-dish sealing film (Diversified Biotech, Dedham, MA, USA, Cat. No. PSEAL-108R), and incubated for 96 h at 26 ± 1°C. After incubation, a minimum of 10 colonies from each mSLAD agar plate were randomly selected and photographed at 2.5× total magnification using a ZEISS Axio Lab.A1 FL-LED microscope (Zeiss, Oberkochen, Germany). This set-up was equipped with a ZEISS Axiocam 105 color camera (Zeiss, Oberkochen, Germany), offering a pixel resolution of 2.2 µm. The images were saved in JPEG format and manually checked, whereafter those deemed of suitable image quality (e.g. absence of blur, no visible media artifacts, not near other colonies) were selected for further image analysis and determination of filamentation degree by the HYPHAEdelity whole colony filamentation analysis pipeline (Britton et al. 2022).

### Image analysis and processing pipeline

**Figure 1.**
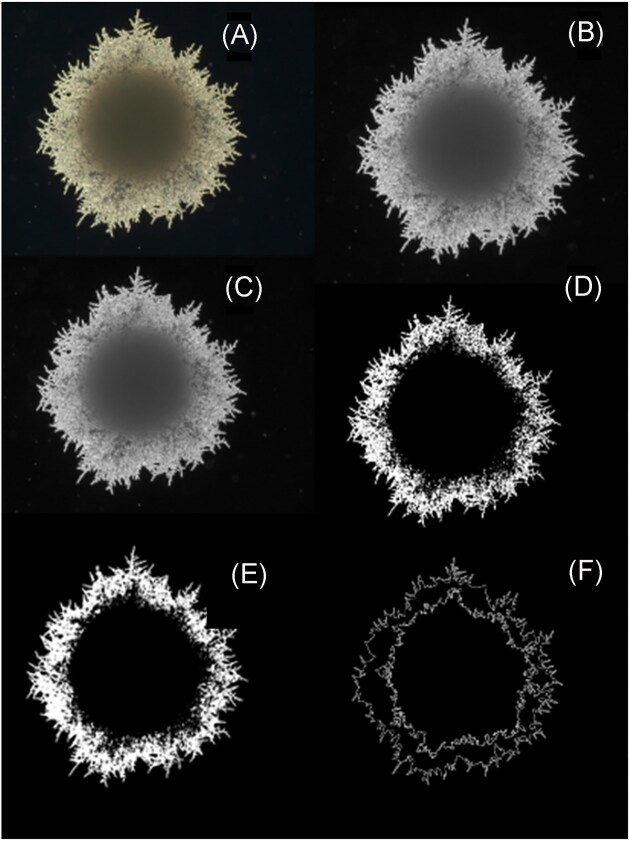
A detailed stepwise schematic illustrating the HYPHAEdelity pipeline for whole colony filamentation analysis. This whole colony image example, captured at 2.5× magnification, illustrates the evaluation of a single colony isolate of *D. bruxellensis* cultured on mSLAD medium. (A) Original image standardized to a resolution of 2560 × 1920, (B) conversion of the original image from the BGR color space to grayscale, (C) application of Gaussian blur filter with a 5 × 5 rectangular kernel, (D) binary conversion with manual threshold configuration, (E) removal of small voids in filamentation boundary, and (F) determination of the *A*_inner_ and *A*_outer_ contour lines.

Image analysis, processing, and filamentation assessment on captured whole colony images were assessed using the HYPHAEdelity analysis pipeline, a publicly accessible two-dimensional whole-colony filamentation measurement tool using a manually set threshold. As described by Britton et al. (2022), whole colony images underwent several steps to ensure quantification precision. Initially, (A) originally images were automatically standardized to a resolution of 2560 × 1920. (B) The images were then converted from the blue–green–red (BGR) color space to grayscale. (C) Subsequently, a Gaussian blur filter with a 5 × 5 rectangular kernel was applied to smooth the images. Following this, (D) a binary conversion was performed, and (E) small voids caused by overlapping filaments and filaments merging into the central colony mass were removed. In the final step, (F) the two most prominent contours within each image were identified: the first, the boundary encompassing all filamentous protrusions outside the central colony mass (designated as *A*_outer_), and the second, the boundary of the central colony mass (designated as *A*_inner_). The normalized growth index value, referred to as the *f-measure*, is effectively the % change of the outer boundary area compared to the inner boundary area. A schematic example of the HYPHAEdelity whole colony filamentation analysis pipeline on one colony isolate of *D. bruxellensis* can be viewed in Fig. 1.

## Results

Table 1 offers the aggregated mean and standard deviations of *f-measures* determined for the *D. anomalous* and *D. bruxellensis* strains across the experimental conditions and replicates. These descriptive statistics were determined using IBM SPSS Statistics v. 28 (Armonk, New York, USA). Prior to mean and standard deviation calculations, multiple outliers were arithmetically determined and excluded based on the Iglewicz and Hoaglin method, utilizing modified *z* scores with a threshold of >3.5 to identify outliers (Hoaglin and Iglewicz 1987). Markedly, in both *Dekkera* strains, a general excitation in pseudohyphal filamentous growth is observed in agar with greater than 10 µM concentrations of 2-phenylethanol and tryptophol, as observed by the increasing mean; however, at the highest concentrations, a decrease in whole colony pseudohyphal growth was observed under specific conditions in certain strains. A photographic example of the morphogenic yeast-to-filamentous transition in *Dekkera* spp., induced by exposure to exogenous aromatic alcohols, is depicted in Supplementary Fig. S1.

**Table 1.** Composite *f-measure* means and standard deviations, after outlier removal, for *D. anomalous* and *D. bruxellensis* filamentation assays under varying experimental conditions. Results reflect filamentation responses to differing concentrations of the fungal quorum-sensing compounds 2-phenylethanol and tryptophol.

	2-Phenylethanol				Tryptophol			
	*n*	*D. anomalous*	*n*	*D. bruxellensis*	*n*	*D. anomalous*	*n*	*D. bruxellensis*
0 µM	47	0.467 ± 0.270	99	0.330 ± 0.217	85	0.310 ± 0.190	88	0.721 ± 0.459
10 µM	46	0.489 ± 0.350	62	0.398 + 0.192	44	0.341 ± 0.197	49	0.723 ± 0.498
50 µM	53	1.109 ± 0.932	106	0.847 ± 0.810	76	0.964 ± 0.663	79	0.856 ± 0.435
100 µM	59	0.969 ± 0.946	91	1.776 ± 1.299	77	1.443 ± 0.996	79	2.085 ± 1.547
200 µM	53	1.570 ± 1.206	77	1.770 ± 1.319	80	0.657 ± 0.442	53	1.373 ± 1.222

Intra-strain differences in *f-measure* means across experimental conditions, the non-parametric Kruskal–Wallis Test was employed. This approach was selected due to the non-uniform distribution of measurements across experimental groups. Statistical analysis was conducted using IBM SPSS Statistics v. 28 (Armonk, New York, USA). The χ²(3) statistics and corresponding *P* values for each strain are detailed in Table 2. Statistical results from the Kruskal–Wallis test indicate the distribution of intra-strain *f-measures* is not equivalent for *D. anomalous* or *D. bruxellensis* under either quorum condition (*P* < .050).

**Table 2.** Kruskal–Wallis test summary for independent samples. The table presents the statistical summary of Kruskal–Wallis test results for *D. anomalous* and *D. bruxellensis* under two quorum experimental conditions. The results indicate that for both strains, at least one treatment significantly differs from another (*P* < .050) regarding stochastic dominance.

	2-Phenylethanol		Tryptophol	
	*D. anomalous*	*D. bruxellensis*	*D. anomalous*	*D. bruxellensis*
*N*	251	435	333	352
χ²(3)	37.598^a^	184.659^a^	156.690^a^	63.968^a^
DF	4	4	4	4
*P* value	<0.001*	0.000*	0.000*	<0.001*

a The test statistic has been adjusted for ties.

* Indicates a statistically significant result (*P* < .050).

A Dunn's test for multiple comparisons was conducted to identify significant differences between paired quorum conditions for the yeast species *D. anomalous* and *D. bruxellensis*. Statistical analysis was conducted using IBM SPSS Statistics v. 28 (Armonk, New York, USA). The test statistic, standard error, standard test statistic, significance value (α = 0.050), and Bonferroni adjusted significance value (α = 0.050) for each paired condition are detailed in Tables 3 and 4. In terms of statistical significance, collectively, the strains of *D. anomalous* and *D. bruxellensis* used in this investigation did not exhibit an excitation of the yeast-to-filamentous transition, indicated by a statistically relevant increase in the *f-measure*, until the exogenous 50 µM concentration of 2-phenylethanol or tryptophol was achieved (sig. *P* < .050). Although the more scrutinous Bonferroni corrected significance value is mildly insignificant for *D. bruxellensis* (adj. sig. *P* = .054) pairwise comparison between 0 and 50 µM treatments; it is our viewpoint that this level of statistical scrutiny is too conservative (Perneger 1998). Meanwhile, regardless of the strain, the lesser concentration of 10 µM resulted in no significant change in filamentation degree compared to the control condition regardless of the quorum compound condition (*P* > .050), which is in line with the threshold concentrations observed in *S. cerevisiae* for exhibiting filamentous social behavior (Chen and Fink 2006).

**Table 3.** Pairwise comparisons of 2-phenylethanol experimental conditions of *D. bruxellensis* and *D. anomalous*. Each row tests the null hypothesis that the paired condition 1 and paired condition 2 distributions are the same. Asymptotic significances (two-sided tests) are displayed. The significance level α = 0.05.

	Paired conditions	Test statistic	Std. error	Std. test statistic	Sig.	Adj. sig.^a^
*D. bruxellensis*	0–10 µM	−33.535	20.361	−1.647	0.100	0.995
	0–50 µM	−89.473	17.571	−5.092	<0.001*	0.000*
	0–100 µM	−199.641	18.257	−10.935	0.000*	0.000*
	0–200 µM	−198.338	19.103	−10.383	0.000*	0.000*
	10–50 µM	−55.938	20.100	−2.783	0.005*	0.054
	10–100 µM	−166.106	20.703	−8.023	<0.001*	0.000*
	10–200 µM	−164.802	21.452	−7.682	<0.001*	0.000*
	50–100 µM	−110.168	17.966	−6.132	<0.001*	0.000*
	50–200 µM	−108.864	18.825	−5.783	<0.001*	0.000*
	100–200 µM	1.304	19.466	0.067	0.947	1.000
*D. anomalous*	0–10 µM	1.707	15.441	.111	0.912	1.000
	0–50 µM	−52.442	13.968	−3.754	<0.001*	0.002*
	0–100 µM	−42.539	14.031	−3.032	0.002*	0.024*
	0–200 µM	−67.970	13.968	−4.866	<0.001*	0.000*
	10–50 µM	−54.149	15.057	−3.596	<0.001*	0.003*
	10–100 µM	−44.246	15.115	−2.927	0.003*	0.034*
	10–200 µM	−69.677	15.057	−4.628	<0.001*	0.000*
	50–100 µM	9.903	13.608	0.728	0.467	1.000
	50–200 µM	−15.528	13.543	−1.147	0.252	1.000
	100–200 µM	−25.431	13.608	−1.869	0.062	0.616

a Significance values have been adjusted using the Bonferroni correction for multiple tests.

* Indicates a statistically significant result (*P* < .050).

**Table 4.** Pairwise comparisons of tryptophol experimental conditions of *D. bruxellensis* and *D. anomalous*. Each row tests the null hypothesis that the paired condition 1 and paired condition 2 distributions are the same. Asymptotic significances (two-sided tests) are displayed. The significance level α = 0.05.

	Paired conditions	Test statistic	Std. error	Std. test statistic	Sig.	Adj. sig.^a^
*D. bruxellensis*	0–10 µM	11.794	17.996	0.655	0.512	1.000
	0–50 µM	−32.807	15.608	−2.102	0.036*	0.356
	0–100 µM	−108.130	15.608	−6.928	<0.001*	.000*
	0–200 µM	−56.129	17.548	−3.199	0.001*	0.014*
	10–50 µM	−44.602	18.504	−2.410	0.016*	0.159
	10–100 µM	−119.925	18.504	−6.481	<0.001*	0.000*
	10–200 µM	−67.923	20.167	−3.368	<0.001*	0.008*
	50–100 µM	−75.323	16.191	−4.652	<0.001*	0.000*
	50–200 µM	−23.322	18.068	−1.291	0.197	1.000
	100–200 µM	52.001	18.068	2.878	0.004*	0.040*
*D. anomalous*	0–10 µM	−11.166	17.880	−0.624	0.532	1.000
	0–50 µM	−127.305	15.199	−8.376	0.000*	0.000*
	0–100 µM	−162.947	15.146	−10.758	0.000*	0.000*
	0–200 µM	−81.563	17.052	−4.783	<0.001*	0.000*
	10–50 µM	−116.139	18.237	−6.368	<0.001*	0.000*
	10–100 µM	−151.781	18.194	−8.342	0.000*	0.000*
	10–200 µM	−70.397	19.809	−3.554	<0.001*	0.004*
	50–100 µM	−35.641	15.567	−2.290	0.022*	0.220
	50–200 µM	45.743	17.427	2.625	0.009*	0.087
	100–200 µM	81.384	17.381	4.682	<0.001*	0.000*

a Significance values have been adjusted using the Bonferroni correction for multiple tests.

* Indicates a statistically significant result (*P* < .050).

Moreover, increasing concentrations of the exogenous fungal quorum compound 2-phenylethanol in *D. bruxellensis* resulted in a statistically significant increase in whole colony filamentation, as indicated by the *f-measure*. Comparisons between 10 and 50 µM (*P* = .016) and between 50 and 100 µM (*P* < .001) showed clear increases. However, no significant difference was observed when comparing 100–200 µM (*P* = .947). Similarly, *D. anomalous* demonstrated a significant increase in filamentation between 10 and 50 µM (*P* < .001), but no further significant change at higher concentrations. As anticipated, the data in both cases indicate a limit to the extent of the social response that can be triggered by a quorum compound. However, a notable disparity is evident between the two strains in question.

Likewise, similar results appear for the quorum conditions with tryptophol, with increasing whole colony filamentation occurring between 10 and 50 µM (*P* < .000), 50 and 100 µM (*P* < .022) for *D anomalous* and 10 and 50 µM (*P* < .001), 50 and 100 µM (*P* = .022), and 10 and 50 µM (*P* = .016), 50 and 100 µM (*P* < .001) for *D. bruxellensis*. However, in comparisons between the 100 and 200 µM concentrations, a statistically significant decrease in filamentation was observed for both strains (*D. anomalous, P* < .001; *D. bruxellensis, P* = .004), possibly due to toxicity at higher concentrations.

## Discussion

The present study demonstrates that quorum-sensing compounds, specifically 2-phenylethanol and tryptophol, governs the increase in yeast-to-filamentous transition in two *Dekkera* spp., namely *D. anomalous* and *D. bruxellensis* at concentrations above 50 µM. Notably, the precise threshold for initiating the morphogenic transition is likely lower, existing within the range of greater than 10 µM and less than 50 µM. These experimental results are consistent with the literature on other ascomycete yeasts, such as *S. cerevisiae* and *C. albicans*, where strains also display enhanced filamentous in response to quorum-sensing signals (Alem et al. 2006, Chen and Fink 2006, Lenhart et al. 2019, Winters et al. 2022, Britton et al. 2023). The conservation of the filamentous social response across *S. cerevisiae, C. albicans*, and *Dekkera* spp. further suggests a broader reliance on quorum sensing as a regulatory mechanism for coordinating diphasic social behaviors in fungi exists, particularly with members belonging to the phylum *Ascomycota*.

The implications of understanding the regulatory mechanisms behind the yeast-to-filamentous transition, a feature associated with increased adhesive properties to inert surfaces, could offer potential future strategies to mitigate *Dekkera* spp., not only in beverage industries but also in any context where the presence of these yeast poses economic risks. On the contrary, this insight could also be used to encourage the establishment of *Dekkera* spp., in environments where the establishment of their biofilms is desirable, such as in traditional sour beers (Verachtert and Derdelinckx 2014, Shayevitz et al. 2020).

In the future, additional research on unraveling the molecular mechanisms underlying quorum sensing in *Dekkera* species, particularly in wild-type and industrially relevant strains, is warranted. A deeper understanding of which genes, or gene clusters, are regulated by quorum sensing could reveal a range of other social behaviors, such as those affecting nutrient assimilation, exoenzyme production, metabolite production, and growth rate, that contribute to food and beverage spoilage. This expanded focus could shed light on new aspects of *Dekkera* biology that have not been fully explored and could pave the way for the development of innovative strategies aimed at mitigating the detrimental effects of *Dekkera* in commercial production environments.

## Supplementary Material

fnae105_Supplemental_File
