# Supplementary material for: Excitation of filamentous growth in Dekkera spp. by quorum sensing aromatic alcohols 2-phenylethanol and tryptophol
Source: FEMS Microbiol Lett. 2024 Dec 5;372:fnae105. doi: 10.1093/femsle/fnae105 (PMC11719618; doi:10.1093/femsle/fnae105)
Supplement: fnae105_Supplemental_File [file fnae105_supplemental_file.docx]

**SUPPLEMENTAL FIGURE S1.**

**
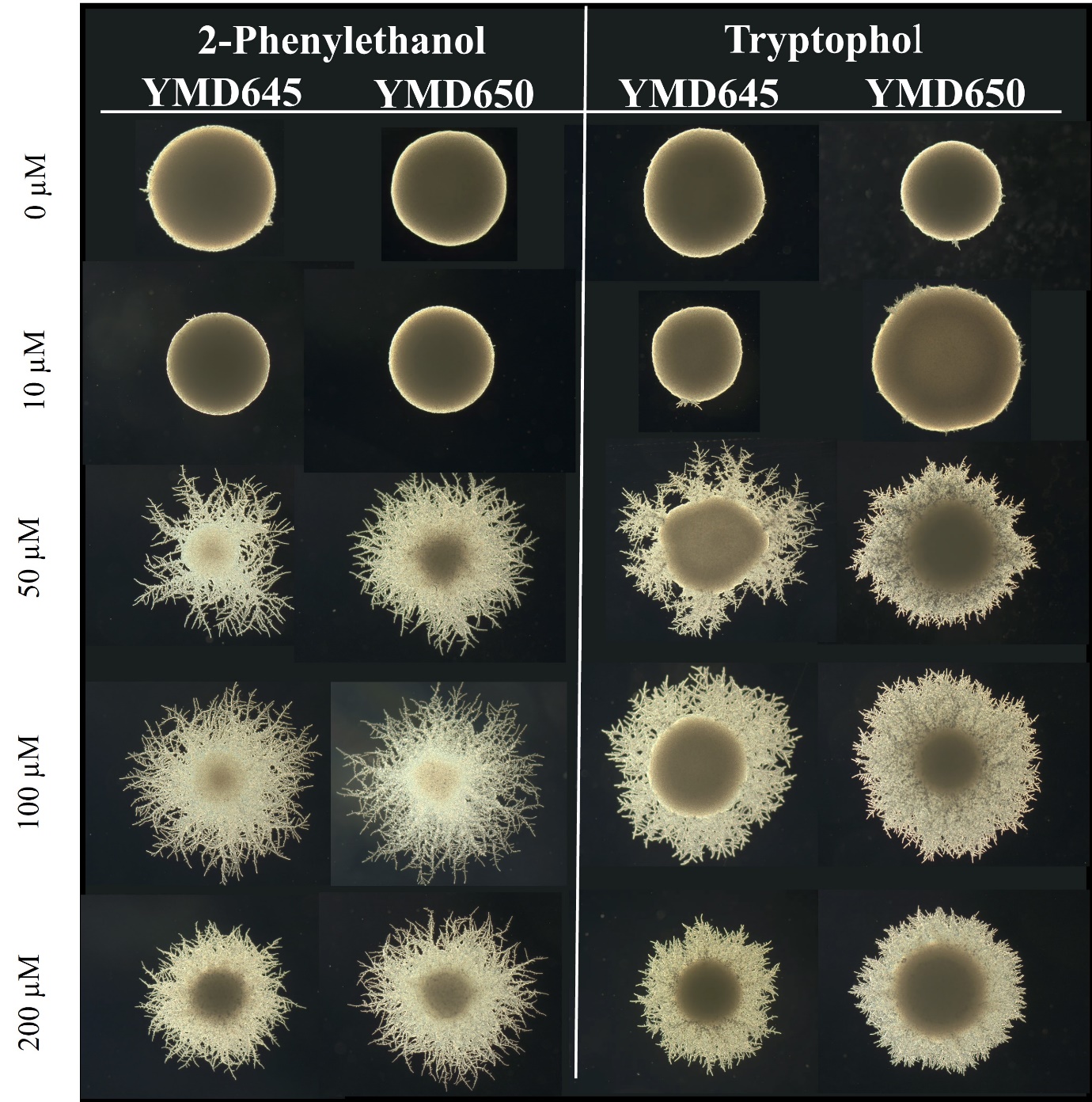
**

**Figure S1**. **Induction of Yeast-to-Filamentous Morphogenesis in Dekkera spp. upon Exposure to Exogenous 2-Phenylethanol and Tryptophol.** This figure presents photographic examples illustrating the colony morphologies of industrial strains of *Dekkera anomalus* (YMD645) and *Dekkera bruxellensis* (YMD650) cultured on mSLAD in response to increasing concentrations of the aromatic alcohols 2-phenylethanol and tryptophol. The transition from yeast-like to filamentous growth is visually captured, highlighting the morphological changes induced by these compounds.
